# Antioxidant Properties of Aminoethylcysteine Ketimine Decarboxylated Dimer: A Review

**DOI:** 10.3390/ijms12053072

**Published:** 2011-05-12

**Authors:** Alberto Macone, Mario Fontana, Marco Barba, Bruno Botta, Mirella Nardini, Francesca Ghirga, Andrea Calcaterra, Laura Pecci, Rosa Marina Matarese

**Affiliations:** 1 Department of Biochemical Sciences, Sapienza, University of Rome, Piazzale Aldo Moro 5, 00185, Rome, Italy; E-Mails: alberto.macone@uniroma1.it (A.M.); mario.fontana@uniroma1.it (M.F.); laura.pecci@uniroma1.it (L.P.); 2 Department of Chemistry and Technology of Drugs Sapienza, University of Rome, Piazzale Aldo Moro 5, 00185, Rome, Italy; E-Mails: marco.barba@uniroma1.it (M.B.); bruno.botta@uniroma1.it (B.B.); francesca.ghirga@uniroma1.it (F.G.); andrea.calcaterra@uniroma1.it (A.C.); 3 National Research Institute on Food and Nutrition Via Ardeatina, 546, 00178 Rome, Italy; E-Mail: nardini@inran.it

**Keywords:** sulfur-containing antioxidants, aminoethylcysteine ketimine decarboxylated dimer, reactive oxygen species, reactive nitrogen species

## Abstract

Aminoethylcysteine ketimine decarboxylated dimer is a natural sulfur-containing compound detected in human plasma and urine, in mammalian brain and in many common edible vegetables. Over the past decade many studies have been undertaken to identify its metabolic role. Attention has been focused on its antioxidant properties and on its reactivity against oxygen and nitrogen reactive species. These properties have been studied in different model systems starting from plasma lipoproteins to specific cellular lines. All these studies report that aminoethylcysteine ketimine decarboxylated dimer is able to interact both with reactive oxygen and nitrogen species (hydrogen peroxide, superoxide anion, hydroxyl radical, peroxynitrite and its derivatives). Its antioxidant activity is similar to that of Vitamin E while higher than other hydrophilic antioxidants, such as trolox and *N*-acetylcysteine.

## 1. Introduction

Oxygen is the key molecule which enables aerobic metabolism in living organisms. However, due to its high reactivity, it is also able to damage bio-molecules by producing reactive oxygen species (ROS) [1]. For this reason living organisms have developed a large and complex network of antioxidant molecules and enzymes, able to protect cellular components such as nucleic acids, proteins and lipids from oxidative damage.

According to a general definition, antioxidants can slow down or prevent the oxidation of other molecules by removing free radical intermediates. Cellular antioxidants can mainly act in two ways: (i) preventing these reactive species from being formed, or (ii) inactivating them before they are able to damage cellular components [2–5]. ROS production occurs physiologically during aerobic metabolism and the main role of the antioxidant network present within the cell is to buffer their overproduction, by keeping them at a level where their physiological role can be carried out (*i.e*., redox signaling) [6]. An imbalance of the antioxidant system may cause severe cellular damage and lead to oxidative stress condition, which is often involved in the pathogenesis of important diseases, such as cancer and atherosclerosis. This imbalance is also implicated in other pathological conditions (such as malaria and rheumatoid arthritis) and could play a role in neurodegenerative diseases and ageing processes [7].

In the last decades, research has been focused on prevention of oxidative damage. The molecular mechanisms involved in the radical scavenging activity of many natural antioxidants and the role they have in human health have been extensively studied. A lot of attention has been focused on dietary antioxidants (α-tocopherol, β-carotene, ascorbic acid), as they may act together with endogenous antioxidant metabolites and enzymes (superoxide dismutase, glutathione peroxidase, catalase) in reducing ROS level. Several epidemiological studies have already highlighted that a high intake of plant products rich in antioxidants is associated with a reduced risk of a number of severe chronic diseases, such as atherosclerosis and cancer. The protection that fruits and vegetables provide against several diseases has been attributed to various antioxidants present in these species, such as vitamin C, vitamin E, α-tocopherol, β-carotene and polyphenolic compounds [8–11].

Related studies have also shown that many of these antioxidant compounds show anti-inflammatory, anti-atherosclerotic, antimutagenic, anticarcinogenic, antibacterial, or antiviral activities to a greater or lesser extent [12–15].

In recent years the work of our research group has been focused on a small group of natural sulfur-containing iminoacids deriving from l-cystathionine, l-lanthionine and (*S*)-2-aminoethyl-l-cysteine [16]. All of them have been identified in biological tissues and fluids but, until now, their biological function is still unclear [17–24]. Among these compounds, aminoethylcysteine ketimine decarboxylated dimer was also detected in many edible vegetables [25] and, while a physiological role has not yet been described for this molecule, its antioxidant properties have been extensively explored and are described in this review.

## 2. Sulfur-Containing Antioxidants

Sulfur plays a critical role in biology, it is widely distributed among living organisms and, particularly, is found in many peptides, proteins and low molecular weight compounds [26,27]. Among the sulfur species found in plants, bacteria, fungi and animals, there are many agents with unique chemical and biochemical properties, which are linked to redox processes, metal binding and catalytic reactions. The antibiotic and anticarcinogenic properties of sulfur compounds such as thiols, thiosulfinates, thiosulfonates, isothiocyanates, sulfoxides, sulfones, sulfinates and polysulfides make them interesting from a pharmacological perspective [28]. The full impact of sulfur in biological systems becomes evident when one considers the diversity of sulfur species and the reactions in which sulfur is involved. These reactions are the result of: (i) the range of different oxidation states sulfur can occupy *in vivo*; (ii) the abundance of different chemotypes possible for each oxidation state; (iii) the various chemical properties that each chemotype exhibits in addition to redox activity, *i.e*., electrostatic interactions, hydrogen bonding, acid-base properties, nucleophilicity and electrophilicity, metal-binding and catalysis. Among these properties, redox-activity, metal binding and nucleophilic substitution are the mechanism most frequently employed by cells to remove oxidative stressors, adventitious metal ions and toxic substances [26,27].

The best investigated sulfur-containing antioxidant compounds are, cysteine, methionine, taurine, glutathione, lipoic acid, mercaptopropionylglycine and *N*-acetylcysteine [29].

**Figure 1.**
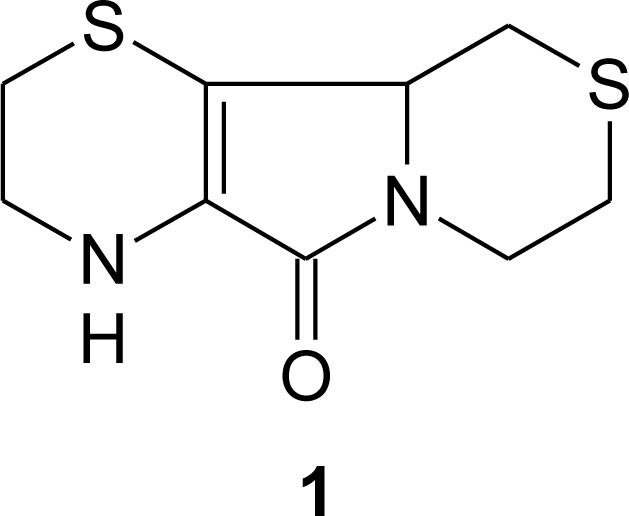
Aminoethylcysteine ketimine decarboxylated dimer.

In recent years, increasing attention has been focused on the discovery of new physiological sulfur compounds that show antioxidant activities. Among these, a very interesting one is aminoethylcysteine ketimine decarboxylated dimer (Figure 1), which is a member of a group of eterocyclic ketimines containing sulfur and nitrogen arising from the metabolism of l-cystathionine, l-lanthionine and (*S*)-2-aminoethyl-l-cysteine [16]. Many of these ketimines and their derivatives have been found in detectable amounts in mammalian tissues and fluids [17–24].

## 3. Aminoethylcysteine Ketimine Decarboxylated Dimer

The chemical synthesis of **1** was first achieved by Hermann and coworkers in 1961 in very good yields starting from cysteamine and bromopyruvic acid [30].

Over the past several years, this molecule has been detected in many mammalian tissues and fluids (human plasma, 2–3 μM, [22], human urine [23] and in bovine cerebellum, 0.6–1 nmol/g wet weight [24]). Recently it was also identified in the brain of cysteamine-treated rats [31]. These findings seem particularly important because they allow formulating new hypotheses on the metabolic origin of this molecule. According to Pinto and coworkers [31], **1** cannot be detected in the brain of rats that are not fed a cysteamine-supplemented diet, suggesting that the brain has the capacity to synthesize **1** when supplied with an adequate amount of this aminothiol.

**Scheme 1.**
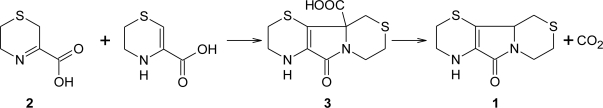
Hypothetical pathway leading to the *in vivo* synthesis of **1.**

In fact, although a metabolic route leading to *in vivo* formation of **1** has not been identified yet, one of the current hypotheses is that it can be formed *in vivo* from the dimerization of aminoethylcysteine ketimine (**2**), followed by a spontaneous decarboxylation step [20] (Scheme 1).

Compound **2** can be endogenously synthesized starting from cysteamine and serine in a two-steps biosynthetic path involving cystathionine β-synthase and glutamine transaminase [32].

As **1** has been detected in many common Mediterranean edible vegetables (where it is present at a concentration range of nmol/g plant material), it can be alternatively considered a vitamin-like compound being introduced in the diet.

Besides efforts to detect this molecule in biological samples, over the past decade many studies have been undertaken to identify its metabolic role. Attention has been focused on its antioxidant properties and on its reactivity against oxygen and nitrogen reactive species. These properties have been studied in different model systems starting from plasma lipoproteins to specific cellular lines.

All these studies report that **1** is able to interact with reactive oxygen and nitrogen species (hydrogen peroxide, superoxide anion, hydroxyl radical, peroxynitrite and its derivatives) [33–36] in all the tested models. In addition, in order to understand the molecular mechanism underlying its antioxidant properties, research efforts have been focused on the oxidation products of **1**. Until now three different products have been isolated and characterized [37–39], while several others need further analyses to be identified. As expected, sulfur plays a key role in the antioxidant properties of **1**. Each sulfur atom can be oxidized to sulfoxide and subsequently to sulfone, leading to the formation of several oxidized species. In addition, a dimeric form and a dehydrogenated product of **1** were also detected, suggesting that the molecular architecture is as important as the presence of sulfur groups.

### 3.1. Aminoethylcysteine Ketimine Decarboxylated Dimer as Scavenger of Reactive Oxygen Species

**Scheme 2.**
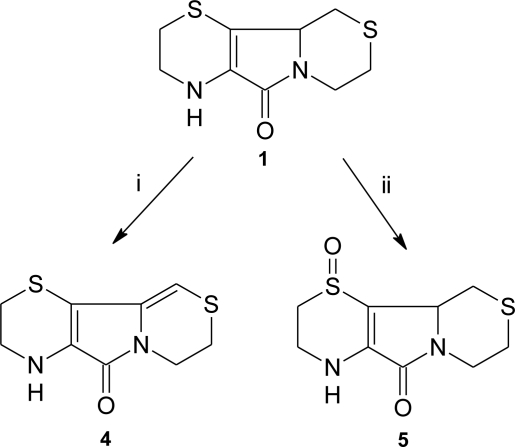
Oxidation products of aminoethylcysteine ketimine decarboxylated dimer. *Reagents and conditions*: (i) CuCl_2_/*t-*BOOH or 2,2′-azo-bis-2-amidinopropane-HCl, (ii) hydrogen peroxide.

Various processes inside the cell produce reactive oxygen species (ROS). Some of the most common ROS are hydrogen peroxide (H_2_O_2_), superoxide ion (O_2_^−^), hydroxide and hydroxyl ions (OH^−^ and OH- respectively). Compound **1** is able to quench ROS leading to the formation of different oxidative products. In Scheme 2 two oxidation products of **1**, which have been isolated and characterized so far, are shown.

Compound **4**, which shows an additional unsaturation with respect to **1**, may arise from an oxidative dehydrogenation path that involves the carbons 10 and 10a of the tricyclic structure. This molecule is formed *in vitro* in the presence of CuCl_2_/*tert*-butyl hydroperoxide (*t-*BOOH) or 2,2′-azo-bis-2-amidinopropane hydrochloride [37]. Compound **5** is produced when **1** reacts with hydrogen peroxide [38].

Several other species have been identified by GC-MS but not yet unambiguously characterized. Among these, the chromatographic analysis shows the presence of at least four different oxidation products, whose mass and fragmentation patterns suggest the formation of **1**-sulfone, **4**-sulfoxide, **4**-sulfone and hydroxyl-**1**, on the basis of that reported by Pecci *et al*. [38].

As already mentioned, endogenous ROS are known to play an important role in ethiopathogenesis of some common and severe chronic diseases, such as atherosclerosis.

Besides the *in vitro* studies carried out to test the quenching activity of **1** towards ROS, *ex vivo* assays on isolated human low-density lipoproteins (LDL) were performed. It is known that oxidation of LDL is an important event implicated in the pathogenesis of atherosclerosis [40]. Oxidized LDL that are no longer recognized by the LDL receptor of the macrophages, are internalized in these cells, via the scavenger receptor pathway, leading to the formation of foam cells [41,42]. The resistance of LDL to oxidative modification is linked to its fatty acids composition and to circulating levels of antioxidant compounds [43].

Compound **1** has already been found to be associated to plasma lipoproteins in humans [22], while another study demonstrated its efficacy in inhibiting the copper-catalyzed oxidation of human LDL [44]. Cu^2+^ catalyzes the lipoprotein oxidation in two main ways: (i) at the protein moiety level [45]; (ii) at the lipid moiety level, via decomposion of pre-existing lipid hydroperoxides and generation of peroxyl- and alkoxyl radicals, which initiate lipid peroxidation [46–48]. Since **1** shows scavenging activity against peroxyl (or alkoxyl) radicals, the main propagating species involved in the LDL lipoperoxidation, it seems reasonable to hypothesize that this could be the possible molecular protection mechanism carried out by **1** against LDL oxidation. The biological relevance of this study is that AECK-DD is active at concentrations comparable to those found in human plasma [22]. For this reason AECK-DD antioxidant properties were also tested on a human U937 monocytic cell line in the presence of the oxidant *t-*BOOH [49].

In fact, it has been recognized that monocyte-endothelium adhesion is a crucial early event in atherogenesis and that plasma antioxidants can prevent or reduce it [50,51]. U937 is a well characterized cell line and the response of this cell line to various inflammatory agents has been well documented [52,53].

The antioxidant activity of **1** is carried out within the U937 cells, as its uptake has been demonstrated by HPLC-ECD and GC-MS analyses [49,54]. A 24 h treatment with 50 and 250 μM AECK-DD, resulted in the incorporation of 0.10 ± 0.01 and 0.47 ± 0.08 ng AECK-DD × 10 [6] cells respectively [49]. In addition, **1** did not display any cytotoxic effect up to the range of mM concentration in the culture medium. Further, at this concentration level, no pro-apoptotic effect has been observed by DNA fragmentation measurements [49]. Compound **1** in concentration range 4–100 μM protects U937 cells from oxidative injury, as revealed by the higher viability maintained with respect to control cells during *t-*BOOH treatment. It is reported that low concentrations of *t*-BOOH lethally affect cultured cells by a mechanism that is dependent on the cellular lipids peroxidation [55]. Therefore, an association of **1** to cellular membranes due to its hydrophobicity could explain its high efficiency in protecting cells against *t-*BOOH-induced oxidative injury. The antioxidant activity of **1** was compared with other known antioxidants with similar results to that of vitamin E, and higher than other hydrophilic antioxidants tested (trolox and *N*-acetylcysteine). The results obtained by Macone and coworkers [49] indicated that the ability of **1** to protect human monocytic U937 cells from *t*-BOOH-induced oxidative stress seems to be mediated by its ability to maintain both intracellular glutathione levels and a reducing environment inside the cell, and to slow down the onset of lipid peroxidation.

The protective effect of **1** might be due to a direct quenching of free radicals, produced during *t-*BOOH treatment, by **1** itself. Moreover, data indicate that **1** is able to significantly reduce the intracellular level of pro-oxidant species in U937 cells in basal conditions. Such studies demonstrated for the first time an antioxidant action of **1** inside the cells, and its ability to modulate cellular response to oxidative stress. As already mentioned, the concentration of **1** in which its antioxidant activity is carried out, is in the range of that measured in human plasma from healthy subjects in fasting conditions [22]. In addition, due to the presence of **1** in human diet, the physiological concentrations of this molecule in non-fasting conditions may be expected to be even higher than those measured in fasting humans. Therefore it can be suggested that, at the concentrations present in human plasma, **1** can really play a significant role in the modulation of oxidative processes *in vivo*.

### 3.2. Aminoethylcysteine Ketimine Decarboxylated Dimer as Scavenger of Reactive Nitrogen Species

Reactive nitrogen species (RNS) are a family of molecules derived from nitric oxide (^•^NO) [56]. In particular, the product of the diffusion-controlled reaction between nitric oxide (^•^NO) and superoxide anion (O_2_^−^) is peroxynitrite (ONOO^−^/ONOOH), a strong oxidizing and nitrating agent that reacts with several biomolecules [57–63]. Besides its activity as oxidative process modulator, a scavenging activity on peroxynitrite has been described for **1** [36].

**Scheme 3.**
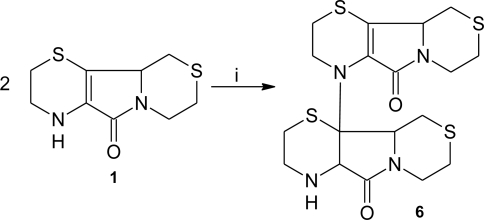
Production of a dimeric form of aminoethylcysteine ketimine decarboxylated dimer in the presence of peroxynitrite. *Reagents and conditions*: (i) peroxynitrite.

Peroxynitrite is an endogenous mediator of various forms of tissue damage in several human pathologies, including neurodegenerative diseases, atherosclerosis, inflammatory and autoimmune diseases. [64,65]. Peroxynitrite is known to mediate oxidation of suitable substrates, either through a direct two-electron mechanism or through an indirect one-electron reaction involving hydroxyl (^•^OH) and nitrogen dioxide (^•^NO_2_) radicals released during peroxynitrite homolysis [59,66–68]. Under physiological conditions, peroxynitrite predominantly reacts with carbon dioxide [69] and the oxidative reactions of peroxynitrite are mediated by (i) the carbonate radical anion (CO_3_^•−^); (ii) ^•^NO_2_ generated by decomposition of the short-lived peroxynitrite-CO_2_ adduct [70–72]. After reaction with peroxynitrite, **1** undergoes oxidative modifications, yielding a dimeric form of the parent compound (**6**) (Scheme 3), which has so far been isolated and characterized using 1D and 2D nuclear magnetic resonanceand ion trap mass spectrometry [39]. The proposed mechanism for the formation of the peroxynitrite-oxidation derivative of **1** involves the radical dimerization of **1** [39].

Peroxynitrite induces lipid peroxidation [73], oxidizes protein and non-protein thiol groups [61,74] and reacts with tyrosine to yield 3-nitrotyrosine [62]. The occurrence of 3-nitrotyrosine is considered the molecular footprint left by reaction of RNS with biomolecules [75,76], while nitrated tyrosine residues are actually considered as biomarkers in a variety of pathophysiological conditions. In addition, peroxynitrite can oxidize free methionine [77] and methionine residues in proteins, e.g., in the α_1_-antiproteinase (α_1_AP), where the oxidation of a critical methionine residue destroys the α_1_AP activity [78]. Peroxynitrite also plays a role in the oxidation of low density lipoprotein [79]. Its generation *in vivo* leads to oxidative modification of blood lipoprotein, which is thought to be a critical event in the development of cardiovascular diseases, including atherosclerosis [40–43].

Tyrosine, when exposed to peroxynitrite at neutral pH, undergoes nitration to form 3-nitrotyrosine. Compound **1** decreases the peroxynitrite-mediated tyrosine nitration in a concentration-dependent manner. In the μM range concentration of **1**, reduction of nitration was higher than 50%. At higher (100 μM) concentration, the nitration is even completely prevented. Compared with other sulfur compounds with established antioxidant activity, **1** showed a protective effect similar to that of glutathioneand *N*-acetylcysteine, but higher than that of methionine [36].

As reported above, peroxynitrite can oxidize methionine residues in proteins. Treatment of α_1_AP with peroxynitrite causes a strong reduction in its elastase inhibitory capacity, as a consequence of the oxidation of a critical methionine [78]. Compound **1** was able to carry out complete protection against α_1_AP inactivation, even at concentrations much lower than those of peroxynitrite. Compound **1** did not show any protective effect if added to α_1_AP preincubated with peroxynitrite, indicating that **1** did not reverse but prevent α_1_AP inactivation by scavenging peroxynitrite and/or its decomposition products [36].

It has been previously reported that peroxynitrite is able to oxidatively modify LDL [79]. LDL preincubation with **1** before peroxynitrite addition clearly demonstrated the effectiveness of **1** in decreasing peroxynitrite-mediated LDL oxidation. Compound **1** was able to prevent the oxidation of LDL almost completely. [36].

These results showed that **1** is a powerful scavenger of peroxynitrite and/or its derived species as it could efficiently protect tyrosine against nitration, α_1_AP against inactivation and LDL against modification. At present these data do not give indications about the mechanism of protection. Compound **1** could act by directly scavenging peroxynitrite or via a combination with reactive intermediates of peroxynitrite decomposition.

## 4. Conclusions

Over the past decade many studies have been undertaken to identify the metabolic role of aminoethylcysteine ketimine decarboxylate dimer, a natural sulfur-containing compound detected in human plasma and urine, mammalian brain and in many common edible vegetables. Attention has been focused on its antioxidant properties and on its reactivity against oxygen and nitrogen reactive species. All the reviewed studies report that aminoethylcysteine ketimine decarboxylate dimer is able to interact both with reactive oxygen and nitrogen species (hydrogen peroxide, superoxide anion, hydroxyl radical, peroxynitrite and its derivatives) protecting human low-density lipoprotein and cultured monocytes against oxidative injury. In these model systems, its antioxidant activity produced similar results to that of Vitamin E and higher than other hydrophilic antioxidant, such as trolox and *N*-acetylcysteine. In addition, **1** is able to quench ROS and RNS leading to the formation of different oxidative products that have been isolated and characterized. The study of several other oxidation products will lead to a better understanding of the molecular mechanism that underlies its powerful action as an antioxidant.
